# Faculty publication trends in a Japanese national university: a diachronic document analysis

**DOI:** 10.1186/s40780-023-00302-0

**Published:** 2023-11-01

**Authors:** Theron Muller, Miho Takano, Nicole Gallagher

**Affiliations:** 1 University of Toyama, 3190 Gofuku, Toyama, 930-8555 Japan; 2 Toyo University, 5-28-20 Hakusan, Bunkyo, Tokyo, 112-8606 Japan

**Keywords:** Pharmaceutical faculty publication, Document analysis, Language of publication, Type of publication, Diachronic analysis

## Abstract

**Background:**

We previously demonstrated that in a Japanese national university’s medical faculty, overall paper publication frequency increased between 1979﻿–1980 and 2017–2018, while original paper publication did not increase. Further, publication language changed from predominantly Japanese to English. However, whether these trends are specific to medicine or representative of other faculties remains unclear.

**Methods:**

We diachronically analyzed annual university library-produced publication reports for four pharmaceutical and three medical units between 1979﻿–1980 and 2019–2020, elucidating how publication frequency, type, and language medium changed.

**Results:**

All publication types increased for the pharmaceutical faculty, from 2.87 per faculty member per year to 10.77. Publication of original papers more than doubled, from 1.06 per faculty member per year to 2.37. This increase was exclusively in English publication, with no publication of Japanese original papers in 2019–2020. This contrasts with medicine, which, while it demonstrated similar increases in all publication types combined, from 4.92 papers per faculty member per year to 12.78, did not demonstrate as striking an increase in total original paper publication (English and Japanese), from 1.21 papers per faculty member per year to 1.30. However, these two faculties observed similar trends in that English largely replaced Japanese original paper publication. That both faculties’ Japanese original paper publication decreased suggests English language original paper publication comes at the expense of publishing in Japanese. Concerning both faculties together, the increase in publishing frequency for all publication types more than tripled from 4.01 to 12.38. This was largely driven by changes in conference paper publication for the pharmaceutical sciences faculty, where English publication increased 2,775% (0.06 to 1.7 papers per faculty member per year) and Japanese language publication 258% (1.33 to 4.77). While conference paper publication did increase for the medical sciences, its change in total publication frequency was largely driven by ‘other’ types of publication, which increased from 0.51 publications per faculty member per year in 1979–1980 to 5.41 in 2019–2020, largely driven by Japanese language publication.

**Conclusion:**

In 2019–2020, pharmaceutical sciences faculty members largely published original papers in English, so postgraduate education should consider the future likelihood of graduates needing to publish in English.

**Supplementary Information:**

The online version contains supplementary material available at 10.1186/s40780-023-00302-0.

## Background

The prevalence of a ‘publish or perish’ culture is by now cliché, with commentary as early as 1950 lamenting the influence that the metrification of knowledge production has on the advancement of science. [1] More recently, language medium of publication has been investigated, with concerns raised about languages other than English being marginalized in scientific communication. [2] Previous studies examining this topic have adopted bibliometrics, which finds increased publication frequency over time. [3] However, it overrepresents English (indexed) journal publication and underrepresents publishing in languages other than English and publication types other than journals, such as books and conference proceedings. [4, 5] For example, a bibliometric study of pharmaceutical sciences/pharmacy education between 1985 and 2021 found 485 papers, all written in English. [6] Thus, non-English language publication remains underrepresented in studies of academic publishing. Other investigative methods, such as surveys of faculty publication practices, tend to indicate little change over time within and outside Japan. [7, 8] In seeking to clarify whether Japan is an exception to global trends toward increasing publication, an earlier preliminary diachronic document analysis of a Japanese university’s medical faculty found that original paper publication frequency was largely unchanged between 1979–﻿1980 and 2017–2018, although original paper publication shifted from majority Japanese publication to majority English publication. [9] Further, we found that publication frequency for all publication types increased considerably. [9] What remains unclear is whether these trends are reflective of the larger faculty or are unique to the medical faculty. While there is some data for increasing Japanese pharmaceutical sciences publishing, it is limited in scope, concerning articles about the field of antimicrobial stewardship [10] and pharmaceutical sciences/pharmacy education. [6] Here we analyzed library publication reports for four of the university’s pharmaceutical sciences units between 1979–﻿1980 and 2019-2020 to answer whether the publishing trends observed for its medical faculty are also characteristic of its pharmaceutical sciences faculty. Note that this study is interested in examining publication trends, with the causes of these trends outside the scope of investigation. We compare our findings for the two faculties and discuss implications for pharmaceutical sciences postgraduate education.

## Methods

We present a diachronic document analysis [9, 11] of language medium of publication and frequency of publication between the years 1979–﻿1980 and 2019–﻿2020 for a Japanese national university’s pharmaceutical and medical faculties. We analyzed annual university library-produced publication reports across four years [12–15], representing two time periods, 1979–﻿1980 and 2019–﻿2020, for four pharmaceutical (Applied Pharmacology, Biopharmaceutics, Chemical Biology, and Pharmaceutical Physiology Biophysical Chemistry) and three medical (Pathology, Internal Medicine, and Biochemistry) units. [9] The years examined start shortly after the university’s founding and finish with the most recent available data. Consistent with our earlier analysis, [9] two-year periods were averaged to minimize the influence of annual variability in publication frequency. The medical faculty units were selected based on the number of faculty, their international composition, compatibility within the available data over time, and to cover research and clinically oriented fields. [9] The pharmacy faculty units were chosen for similar reasons, with the overriding factor being compatibility between the two time periods examined. Averaged across each of the two years examined, for the pharmaceutical faculty this covered 34.5 (62%) faculty members in 1979–1980 and 15 (26%) faculty members in 2019–2020. For the medical faculty, this covered 43 (24%) faculty members in 1979–1980 and 61 (20%) faculty members in 2019–2020. As the faculty compositions have changed between the periods examined and as pharmaceutical education has changed from four years of education to six [16], a simple comparison of the faculty units is not practical. Therefore, publication frequency was calculated per faculty member per year, thereby normalizing the data. Such an analysis is consistent with previous investigations into historical publication trends. [8, 9].

Analyzing the data across the two time periods can elucidate how publication frequency, type, and language medium have changed. By adopting document analysis, a methodological alternative to bibliometric studies, more publication types can be examined than via bibliometric research methods. Further, examining results from the two faculties can explicate whether trends are discipline-specific or present across disciplines.

## Results

Table 1 shows the overall publication frequency per faculty member per year by publication type for each period analyzed and each faculty as well as for both faculties combined. The medical faculty data for 1979–1980 is republished with permission. [9].

**Table 1:** Publication frequency per faculty member per year for pharmaceutical sciences and medical sciences faculties and both faculties combined, 2019–2020 versus 1979–1980

		Pharmaceutical				Medical				Combined			
		1979–1980	2019–2020	△^1^	%△^2^	1979–1980	2019–2020	△	%△	1979–1980	2019–2020	△	%△
Original Papers	JP^3^	0.30		-0.30	-100%	0.81	0.11	-0.70	-86%	0.59	0.09	-0.49	-84%
	EN^4^	0.75	2.37	+ 1.61	+ 214%	0.40	1.19	+ 0.79	+ 201%	0.55	1.42	+ 0.87	+ 156%
	Σ^5^	1.06	2.37	+ 1.31	+ 124%	1.21	1.30	+ 0.09	+ 8%	1.14	1.51	+ 0.37	+ 33%
Books	JP	0.03	0.13	+ 0.10	+ 360%	0.55	0.21	-0.33	-61%	0.32	0.20	-0.12	-38%
	EN		0.13	+ 0.13	N/A	0.01	0.07	+ 0.05	+ 464%	0.01	0.08	+ 0.07	+ 1,124%
	Σ	0.03	0.27	+ 0.24	+ 820%	0.56	0.28	-0.28	-50%	0.32	0.28	-0.05	-14%
Case Reports	JP					0.01	0.13	+ 0.12	+ 1,028%	0.01	0.11	+ 0.10	+ 1,532%
	EN						0.22	+ 0.22	N/A		0.18	+ 0.18	N/A
	Σ					0.01	0.35	+ 0.34	+ 2,931%	0.01	0.28	+ 0.28	+ 4,285%
Reviews	JP	0.04	0.33	+ 0.29	+ 667%	0.51	0.58	+ 0.07	+ 14%	0.30	0.53	+ 0.23	+ 76%
	EN		0.17	+ 0.17	N/A		0.11	+ 0.11	N/A		0.13	+ 0.13	N/A
	Σ	0.04	0.50	+ 0.46	+ 1,050%	0.51	0.70	+ 0.19	+ 36%	0.30	0.66	+ 0.35	+ 117%
Conference Papers	JP	1.33	4.77	+ 3.43	+ 258%	1.87	3.58	+ 1.71	+ 91%	1.63	3.82	+ 2.18	+ 134%
	EN	0.06	1.67	+ 1.61	+ 2,775%	0.24	1.16	+ 0.91	+ 373%	0.16	1.26	+ 1.10	+ 679%
	Σ	1.39	6.43	+ 5.04	+ 362%	2.12	4.74	+ 2.62	+ 124%	1.79	5.07	+ 3.28	+ 183%
Other	JP	0.29	1.07	+ 0.78	+ 268%	0.51	5.11	+ 4.60	+ 900%	0.41	4.32	+ 3.90	+ 945%
	EN	0.06	0.13	+ 0.08			0.30	+ 0.30	N/A	0.03	0.26	+ 0.24	+ 920%
	Σ	0.35	1.20	+ 0.85	+ 245%	0.51	5.41	+ 4.90	+ 957%	0.44	4.58	+ 4.14	+ 944%
All Types	JP	2.00	6.30	+ 4.30	+ 215%	4.27	9.74	+ 5.47	+ 128%	3.26	9.06	+ 5.80	+ 178%
	EN	0.87	4.47	+ 3.60	+ 414%	0.65	3.04	+ 2.39	+ 367%	0.75	3.32	+ 2.57	+ 344%
	Σ	2.87	10.77	+ 7.90	+ 275%	4.92	12.78	+ 7.86	+ 160%	4.01	12.38	+ 8.38	+ 209%

^1^ Change (publications per faculty member per year), ^2^% change, 2019–2020 relative to 1979–1980, ^3^ Japanese, ^4^ English, ^5^ Total

The increase in overall publication frequency for the medical and pharmaceutical sciences faculty members combined is striking; the average publication frequency per faculty member per year more than tripled from 4.01 to 12.38. Pharmaceutical sciences faculty members’ overall and original paper publication frequency both more than doubled, from 2.87 per faculty member per year to 10.77 and from 1.06 to 2.37, respectively. Medical sciences faculty members’ overall publication frequency increased from 4.92 per faculty member per year to 12.78. However, publication of original papers remained relatively unchanged.

The pharmaceutical sciences faculty members’ increases in original paper publication frequency were exclusively in English publication, with no publication of Japanese original papers in 2019–2020. While medical sciences faculty members showed little change in overall original paper publication frequency, publication in Japanese decreased and English increased, like for the pharmaceutical faculty members. Turning to conference papers, pharmaceutical sciences faculty members’ English language publication frequency increased 2,755% (0.06 papers per faculty member per year to 1.61) and Japanese language publication frequency increased 258% (1.33 papers per faculty per year to 4.77). Although the increase in English language publication is more apparent, there were more conference papers published in Japanese than English in both periods examined. Medical sciences faculty members’ conference papers for both languages also increased, although not as dramatically as for pharmaceutical sciences faculty members. Further, the number of Japanese conference papers published in 2019–2020 remained larger than those published in English, like for the pharmaceutical sciences faculty members. Concerning other publications, the increases for medical sciences faculty members were more drastic than for pharmaceutical sciences faculty members. Book publication increased in both languages for pharmaceutical sciences faculty members. However, medical sciences faculty members’ total book publication decreased by 50%. Further, the number of publications in English increased for all publication types for members of both faculties.

## Discussion

Our findings indicate that previously identified medical sciences faculty publication trends toward increased English language publication and increased publication frequency [9] also exhibit for pharmaceutical sciences faculty. Specifically, for both faculties English language original paper publication came at the expense of publishing in Japanese, with striking increases in English and decreases in Japanese. [9] Further, the trends for pharmaceutical sciences were more striking than for the medical sciences. Publication frequency per faculty member increased for both faculties, exhibiting across all publication types for the pharmaceutical sciences, while medical sciences faculty only showed increases in publication frequency for some publication types. This reinforces earlier bibliometric analyses of global trends toward increased publication, [3] suggesting that earlier findings of different publishing trends for Japan [8] relative to global trends toward increased publication [3] are perhaps methodologically flawed. For example, the period between surveys may not have been long enough to identify changes in publication practices or the use of surveys to measure past actions may lead to misleading results. [17] Considering the prestigiousness of journal original paper publication in English, there is likely considerable pressure to publish such articles. [18] However, the metrification of higher education [3] is also evident through increases in other publication types, such as conference and ‘other’ papers, illustrating how 2019–2020 faculty felt more expectations to publish than their 1979–1980 counterparts. Moving forward, this preliminary analysis would benefit from further coverage of the available publication data, including more faculty units and more years of publication for the pharmaceutical and medical sciences faculties. Nevertheless, we feel our analysis is sufficient to demonstrate that the pharmaceutical and medical sciences faculties at this Japanese national university are exhibiting similar broad trends that reflect larger global trends in publishing practices. [3] We further reveal how publication practices outside original papers, typically investigated through bibliometric analysis, [3, 4] have changed, adding further nuance to the picture of Japanese faculty publication practices. Specifically, conference papers and ‘other’ papers increased in frequency for both Japanese and English publication. Regarding postgraduate education, the expectations for 2019–2020 junior faculty to publish in English are likely different from the expectations of 1979–1980 junior faculty. Therefore, pharmaceutical sciences postgraduate education in Japan, especially for those in research tracks, should likely take the prominence of English publication into account. While some efforts have been made to improve students’ understanding of the importance of reading the pharmaceutical literature, [19] helping postgraduate students to understand the importance of producing literature in English appears to also be called for.

## Conclusions

Pharmaceutical and medical sciences faculty both exhibited increased overall publication. However, there were some differences observed between which publication types exhibited increases. For original papers, increases in English language publication came at the expense of Japanese language publication. These trends suggest that publishing in Japan has followed trends like those observed for Anglophone faculty outside of Japan, [3] trends that postgraduate pharmaceutical education should consider as early career researchers may need to publish in English.

### Electronic supplementary material

Below is the link to the electronic supplementary material.

Supplementary Material 1

## Data Availability

All underlying data from this investigation can be accessed through the University of Toyama Repository at https://toyama.repo.nii.ac.jp/. The datasets analyzed in the current study are available from 10.5281/zenodo.8047365.
