# Supplementary material for: Faculty publication trends in a Japanese national university: a diachronic document analysis
Source: J Pharm Health Care Sci. 2023 Nov 1;9:34. doi: 10.1186/s40780-023-00302-0 (PMC10619260; doi:10.1186/s40780-023-00302-0)
Supplement: Supplementary file 1 — Supplementary Material 1 [file 40780_2023_302_MOESM1_ESM.docx]

Thank you for your review of our manuscript. Your comments helped to improve its readability and focus. Please find our point-by-point responses to the reviewers’ comments below. We have also attached a change tracked version of our manuscript along with a ‘clean’ version without any markup.

| Reviewer comment | Author response |
| --- | --- |
| #1 Please confirm that some of the issues that should be included in the discussion are included in the results.  e.g. P19, L17-18, L38-39, etc. | We have moved the summary paragraph and the “This suggests…” sentence from the Results to the Discussion sections of the manuscript as requested. |
| Reviewer #2: The manuscript has been significantly improved, taking into account the comments from the reviewers.  However, there is a need to reevaluate the consistency between the results and discussion sections. Once the minor issues are addressed, I believe this manuscript will be suitable for acceptance. |  |
